# Lauric Acid versus Palmitic Acid: Effects on Adipose Tissue Inflammation, Insulin Resistance, and Non-Alcoholic Fatty Liver Disease in Obesity

**DOI:** 10.3390/biology9110346

**Published:** 2020-10-22

**Authors:** Viswanathan Saraswathi, Narendra Kumar, Thiyagarajan Gopal, Saumya Bhatt, Weilun Ai, Carmen Ma, Geoffrey A. Talmon, Cyrus Desouza

**Affiliations:** 1 Department of Internal Medicine, Division of Diabetes, Endocrinology, and Metabolism, University of Nebraska Medical Center, Omaha, NE 68198, USA; narendra.kumar@unmc.edu (N.K.); thiyagarajan.gopal@unmc.edu (T.G.); saumya.bhatt@unmc.edu (S.B.); weilun.ai@unmc.edu (W.A.); carmenm1221@yahoo.com (C.M.); cdesouza@unmc.edu (C.D.); 2 VA Nebraska-Western Iowa Health Care System, Omaha, NE 68105, USA; 3 Department of Pathology and Microbiology, University of Nebraska Medical Center, Omaha, NE 68198, USA; gtalmon@unmc.edu

**Keywords:** lauric acid, palmitic acid, medium-chain saturated fatty acids, long-chain saturated fatty acids, obesity

## Abstract

**Simple Summary:**

The aim of this study was to compare the effect of palmitic acid (PA), a long-chain fatty acid, and lauric acid (LA), a medium-chain fatty acid, on obesity-related metabolic disorders. We used a mouse model of diet-induced obesity and fed them a modified high fat diet supplemented with 3% PA or LA for 12 wk. An LA diet led to an increase in visceral fat mass with a reduction in inflammation compared to the PA diet. We also noted that PA significantly increased systemic insulin resistance whereas LA showed only a trend towards an increase compared to lean control mice. The expression of a protein involved in muscle glucose uptake was higher in LA-treated mice compared to the PA-treated group, indicating improved muscle glucose uptake in LA-fed mice. Analysis of liver samples showed that hepatic steatosis was higher in both PA and LA-fed mice compared to lean controls. Markers of liver inflammation were not altered significantly in mice receiving PA or LA. Our data suggest that compared to PA, LA exerts less adverse effects on metabolic disorders and this could be due to the differential effects of these fatty acids in fat and muscle.

**Abstract:**

Coconut oil, rich in medium-chain saturated fatty acids (MCSFA), in particular, lauric acid (LA), is known to exert beneficial metabolic effects. Although LA is the most abundant saturated fatty acid in coconut oil, the specific role of LA in altering obesity-related metabolic disorders remains unknown. Here, we examined the effects of supplementing a high fat (HF) diet with purified LA on obesity-associated metabolic derangements in comparison with palmitic acid (PA), a long-chain saturated fatty acid. Male C57BL/6 mice were fed a control chow diet (CD) or an HF diet supplemented with 3% LA (HF + LA) or PA (HF + PA) for 12 wk. Markers of adipose tissue (AT) inflammation, systemic insulin resistance (IR), and hepatic steatosis, were assessed. The body weight and total fat mass were significantly higher in both HF + LA and HF + PA diet-fed groups compared to CD controls. However, the visceral adipose tissue (VAT) mass was significantly higher (*p* < 0.001) in HF + LA-fed mice compared to both CD as well as HF + PA-fed mice. Interestingly, markers of AT inflammation were promoted to a lesser extent in HF + LA-fed mice compared to HF + PA-fed mice. Thus, immunohistochemical analysis of VAT showed an increase in MCP-1 and IL-6 staining in HF + PA-fed mice but not in HF + LA-fed mice compared to CD controls. Further, the mRNA levels of macrophage and inflammatory markers were significantly higher in HF + PA-fed mice (*p* < 0.001) whereas these markers were increased to a lesser extent in HF + LA-fed group. Of note, the insulin tolerance test revealed that IR was significantly increased only in HF + PA-fed mice but not in HF + LA-fed group compared to CD controls. While liver triglycerides were increased significantly in both HF + PA and HF + LA-fed mice, liver weight and plasma markers of liver injury such as alanine aminotransferase and aspartate aminotransferase were increased significantly only in HF + PA-fed mice but not in HF + LA-fed mice. Taken together, our data suggest that although both LA and PA increased AT inflammation, systemic IR, and liver injury, the extent of metabolic derangements caused by LA was less compared to PA in the setting of high fat feeding.

## 1. Introduction

Consumption of a diet rich in saturated fatty acids (SFAs) is associated with an increased risk of obesity and obesity-associated pathologies, in particular, dyslipidemia, insulin resistance (IR) and non-alcoholic fatty liver disease (NAFLD). Although SFAs are linked to these various metabolic disorders, it is also widely accepted that their metabolic effects vary depending on their chain length. For example, while long-chain SFAs (LCSFAs) are known to cause metabolic derangements, medium-chain SFAs (MCSFAs) have beneficial effects in promoting metabolism.

LCSFAs, in particular, palmitic acid (PA), is highly abundant in palm oil [1] and is known to promote obesity and obesity-related metabolic disorders. A high fat diet containing palm oil significantly increased body weight gain as well as hepatic steatosis compared to a high fat diet containing olive oil in mice [2,3]. In addition, a diet rich in SFAs provided by palm oil caused hepatic lipid accumulation in patients with type 2 diabetes [4]. It should be pointed out that adipose tissue (AT) inflammation is an important mediator of obesity-related metabolic disorders including IR and NAFLD [5,6,7] and LCSFAs promote inflammation in AT in obesity [8,9]. 

While the LCSFAs exert adverse effects on metabolic processes, the medium-chain triglycerides favorably alter metabolism. These fatty acids reduce adiposity and increase hepatic thermogenesis in mice [10]. Zacek et al. have shown that an obesogenic diet enriched in MCSFAs prevented hepatic lipid accumulation and lowered markers of IR compared to a diet supplemented with LCSFA [11]. Zicker et al. have shown that a high carbohydrate diet supplemented with coconut oil, which is rich in MCSFAs, promoted lower adiposity and decreased hepatic steatosis in mice compared to a high carbohydrate diet alone [12]. MCSFAs are also effective in reducing body weight and adiposity [13] and in improving exercise endurance in human subjects [14]. It should be pointed out that most of these studies on MCSFAs have used coconut oil as a source of these fatty acids. 

Coconut oil has beneficial effects against cardiovascular disease [15,16]. It is also protective against high fat diet-induced NAFLD in rats and mice [17,18]. However, concerns exist regarding the hypercholesterolemic effects of coconut oil, which may increase the risk for heart diseases [19,20]. The beneficial effects of coconut oil are attributed to lauric acid (LA), an MCSFA, most abundant in coconut oil. However, it should be noted that coconut oil contains other LCSFAs including myristic acid and PA. Therefore, to better assess the beneficial and the potential adverse effects of coconut oil, studies need to be conducted using diets supplemented with individual fatty acids. Here, we sought to determine the role of purified LA in altering obesity-related inflammation, IR, and NAFLD and to compare its effects with PA in altering these disorders. We hypothesize that LA blocks and PA promotes AT inflammation and the development of IR and NAFLD in obesity. 

## 2. Materials and Methods

### 2.1. Mice and Diet 

Six eight-week-old male wild type C57BL/6 mice were purchased from Jackson laboratory and fed a chow diet (CD, Purina Lab Diet #5001) or a modified high fat (HF) diet (Research Diets Inc., New Brunswick, NJ, USA) for 12 wk. The HF diet (Research Diets Inc. #D12451), which provides 20% protein, 35% carbohydrates and 45% fat in terms of calories, was modified to add purified PA or LA. Briefly, 3% of lard, which is a fat source in the regular HF diet, was replaced with 3% PA or LA. Methyl esters of PA and LA were purchased from Nu-Chek (Elysian, MN, USA) and supplemented in the HF diet. PA was originally isolated from palm oil and LA was derived from palm kernel oil. Mice were divided into three groups: (1) chow diet (CD, *n* = 6), (2) palmitic acid diet (PA, *n* = 8), and (3) lauric acid diet (LA, *n* = 8). Mice were fed respective diets for 12 wk and euthanized under anesthesia. Visceral AT (VAT) and liver weight were recorded. All animal care procedures were carried out with approval from the Institutional Animal Care and Use Committee of VA Nebraska-Western Iowa Health Care System (VA-096).

### 2.2. In Vivo Metabolic Tests

Intraperitoneal insulin tolerance test (ITT) was performed 10 wk post-diet. For ITT, mice were fasted for 5 h and injected (i.p.) with insulin (Novo Nordisk, 0.75 units/kg body weight). Changes in blood glucose levels were recorded at various time points. Intraperitoneal glucose tolerance test (GTT) was performed 11 wk post-diet. Mice were fasted for 5 h and injected (i.p.) with glucose (1 g/kg body weight) and changes in blood glucose levels were recorded at various time points. Whole body composition (lean and fat mass) was assessed by an EchoMRI analyzer before sacrifice and mice were euthanized after 5 h fasting.

### 2.3. Ex Vivo Lipolysis Assay

For ex vivo lipolysis, ~100 mg VAT isolated from HF-fed mice were placed in two wells per sample in 12-well culture dishes to determine basal and stimulated lipolysis. Tissue was cut into ~25-mg pieces and incubated at 37 °C in 1.0 mL of Krebs-Ringer buffer with (stimulated lipolysis) or without (basal lipolysis) 10 μM forskolin for 4 h. Because the VAT weight was much lower in CD-fed mice, we did not include this group in the lipolysis assay. Glycerol level was measured in aliquots from incubation buffer using the Glycerol Assay Kit from Cayman (Ann Arbor, MI, USA), and normalized to tissue weight.

### 2.4. Other Metabolic Variables

Blood glucose was measured using the Accu-Chek Aviva glucometer. For this, ~5 µL of blood from the tail vein was placed in the glucose test strip to measure glucose levels. Plasma total cholesterol and TGs were measured using kits from Raichem (Reggio Emilia, Italy) and Thermofisher (Waltham, MA, USA), respectively. Plasma free fatty acids (FFAs) were analyzed using a kit from Wako. The circulating level of free glycerol in the plasma was quantified colorimetrically using the Cayman’s glycerol assay kit. Plasma samples diluted with water (1:2) were used in the 96-well plate and the amount of glycerol present in the plasma was determined. Plasma alanine aminotransferase (ALT) and aspartate aminotransferase (AST), markers of liver injury, were determined using Vitros Clinical Chemistry Analyzer.

### 2.5. Liver Lipid Analysis

The levels of triglycerides along with their fatty acid profile in liver samples were determined by gas chromatography at the Lipid Core Laboratory of Vanderbilt University, as we described previously [21]. 

### 2.6. Real-Time PCR Analysis

Total RNA was isolated from VAT, gastrocnemius muscle, and liver using Trizol. The reverse transcription mix to convert RNA to cDNA consisted of 1× iScript reverse transcription supermix (Bio-Rad Laboratories, Herculus, CA, USA) and 200 ng RNA in a 20 µL reaction. The cDNA synthesis was carried out in the thermocycler at 25 °C for 5 min., 42 °C for 30 min., and 85 °C for 5 min. The real-time PCR reaction was carried out using 1× iQ Supermix (Bio-Rad Laboratories), 2 µL cDNA, and 1× Taqman primer-probes from Applied Biosystems (Table 1) in a 20 µL reaction. PCR amplifications were always performed in duplicate wells, using the universal temperature cycles: 3 min at 95 °C, followed by 40 two-temperature cycles (10 s at 95 °C and 30 s at 60 °C). A ΔΔCT method was used to calculate the gene expression and the values were normalized to 18S. 

### 2.7. Histology

Liver tissues in cassettes were fixed in 10% formalin for 3 days. The cassettes were then transferred to 70% ethanol. Tissues were embedded in paraffin and 4-µm sections were cut. We performed hematoxylin and eosin (H&E) staining to assess liver pathology. Liver sections were reviewed by Dr. Geoffrey Talmon, a board-certified anatomic pathologist to evaluate the degree of steatosis, inflammation, ballooning, and fibrosis. Slides were scored from 0–4 depending on the degree of injury as evident from these markers. The pictures were taken at 20× magnification using a Nikon eclipse 80i inverted microscope.

MCP-1 in liver and VAT sections was detected by immunohistochemistry. For this, deparaffinized tissue sections were incubated overnight with anti-MCP1 primary antibody (Invitrogen, Waltham, MA, USA) at 4 °C. Sections were then incubated for 1 h with biotinylated secondary antibody (Vector IHC kit PK-4000) at room temperature. Vectastain ABC reagent (Avidin-Biotinylated HRP) (Vector Laboratories, Burlingame, CA, USA) was added for 30 min followed by incubation with peroxidase substrate (DAB, Vector SK-4100) until color developed. Sections were counterstained with hematoxylin. Pictures were taken at 20× magnification using a Nikon eclipse 80i inverted microscope. 

Immunofluorescence analysis was performed to detect IL6 in VAT and GLUT-4 and CPT-1 in skeletal muscle sections. After antigen retrieval, VAT sections were treated with anti-IL-6 primary antibody. After washing, sections were incubated with the Signal Stain boost IHC detection reagent containing rabbit HRP-conjugated secondary antibody (Cell Signaling Tech, Beverly, MA, USA). After washing, sections were incubated with alexa fluor 488 tyramide reagent (Thermo Fisher Scientific, Waltham, MA, USA). For GLUT-4 and CPT-1 staining, after antigen retrieval, muscle sections were incubated with respective primary antibodies followed by incubation with fluorescent-labeled secondary antibodies. Nuclei were stained with DAPI and pictures were taken at 20× magnification using a Nikon eclipse 80i inverted microscope.

### 2.8. Assessment of Hepatic Oxidative Stress 

Lipid peroxidation was assessed by quantifying thiobarbituric acid-reactive substances (TBARS). Glutathione (GSH) depletion is a marker of oxidative stress and therefore, we also measured hepatic glutathione content. TBARS and GSH were determined using kits from Cayman Chemicals (Ann Arbor, MI, USA).

### 2.9. Enzyme-Linked Immunosorbent Assay (ELISA)

The circulating level of plasma adiponectin was measured by the Quantikine Mouse Adiponectin/Acrp30 ELISA kit from R&D Systems (Minneapolis, MN, USA). Plasma samples were diluted 1:2000 as per the manufacturer’s instructions and adiponectin levels were determined by the quantitative sandwich enzyme immunoassay. The color intensity was measured at 450 nm.

### 2.10. Statistical Analysis

Results are presented as mean ± SEM. Statistical significance was determined by the one-way analysis of variance followed by Tukey’s post-hoc analysis. For differences in blood glucose levels during ITT, a two-way analysis of variance (time and treatment) was performed and statistical significance was determined using Tukey’s post-hoc test. The difference between basal and stimulated lipolysis was determined by Student’s *t*-test. Graph-Pad Prism software was used to determine statistical significance (*p* < 0.05 was considered significant). 

## 3. Results

### 3.1. LA Increased VAT Mass Compared to PA without Changes in Total Fat Mass

**Figure 1:**
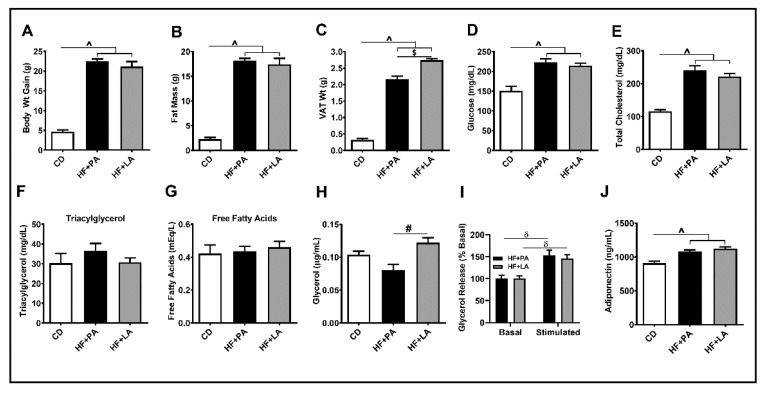
Effect of HF diet supplemented with PA or LA on body weight, fat mass, lipolysis, and systemic metabolic variables in mice. The body weight of mice was recorded at baseline and at 12 wk post-diet and the difference is shown (**A**). The total fat mass was measured in live mice at 12 wk post-diet by an EchoMRI body composition analyzer (**B**). VAT mass was measured after euthanasia (**C**). Blood glucose (**D**) and plasma lipids such as total cholesterol (**E**) and triglycerides (**F**) were assessed. As markers of adipose tissue (AT) lipolysis, plasma free fatty acids (**G**) and glycerol (**H**) were measured. Basal and stimulated lipolysis was assessed ex vivo in VAT from HF-fed mice (**I**) and plasma adiponectin levels were determined by ELISA (**J**). Values are expressed as mean ± SEM of 6–8 samples in each group. ^ *p* < 0.001 versus CD; ^#^ *p* < 0.01; ^$^ *p* < 0.001 versus HF + PA; ^δ^ *p* < 0.01 versus basal. VAT, visceral adipose tissue; CD, chow diet; HF + PA, high fat + palmitic acid; HF + LA, high fat + lauric acid.

As shown in Figure 1A, the body weight of mice increased significantly in HF + PA and HF + LA-fed mice compared to CD-fed controls (*p* < 0.001). The total fat mass was also significantly higher in HF + PA and HF + LA-fed groups compared with CD controls (*p* < 0.001) (Figure 1B). Similarly, the VAT mass increased significantly in both HF + PA and HF + LA-fed mice (*p* < 0.001) (Figure 1C). Interestingly, the HF + LA-fed mice exhibited a significantly higher VAT mass compared to HF + PA-fed mice (*p* < 0.001). Fasting blood glucose and plasma total cholesterol (Figure 1D,E) were significantly higher in both HF + PA and HF + LA-fed mice compared to CD controls (*p* < 0.001) whereas the plasma triacylglycerol did not change significantly (Figure 1F). To determine whether the increase in VAT mass in HF + LA-fed mice was due to reduced AT lipolysis, we measured plasma FFAs and glycerol. The levels of FFAs did not differ among groups (Figure 1G) whereas plasma glycerol levels were significantly higher in HF + LA-fed mice compared to HF + PA-fed mice (*p* < 0.01) (Figure 1H). We also measured basal and stimulated lipolysis in VAT explants. We noted that the glycerol release upon forskolin stimulation was significantly higher (*p* < 0.01) in both HF + PA and HF + LA-fed mice compared to their respective basal levels. On the other hand, no difference was noted between HF + PA and HF + LA-fed mice (Figure 1I). Finally, we determined plasma adiponectin levels by ELISA. As shown in Figure 1J, adiponectin levels rose significantly (*p* < 0.001) in both HF + PA and HF + LA-fed mice compared to CD controls. Together, these data show that supplementation with LA leads to increased VAT mass and AT lipolysis without many changes in other metabolic variables compared to PA supplementation. 

### 3.2. A High Fat Diet Supplemented with LA Promoted Less AT Inflammation Compared to PA

**Figure 2:**
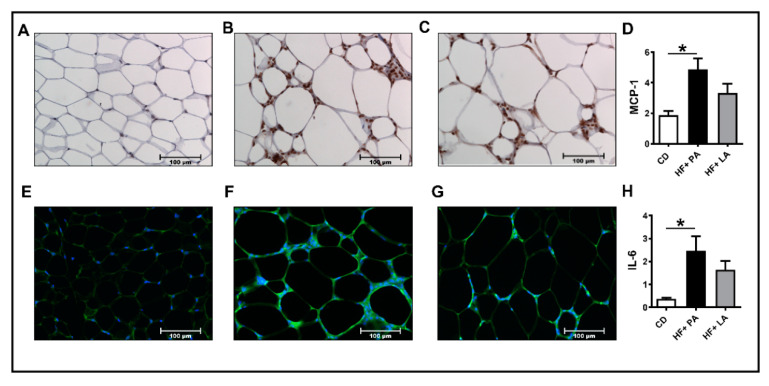
Effect of HF diet supplemented with PA or LA on VAT inflammation. Immunohistochemistry was performed to detect MCP-1 in the visceral adipose tissue (VAT) sections from CD (**A**), HF + PA (**B**), and HF + LA-fed mice (**C**). Representative images at 20× magnification are shown. The intensity of MCP-1 staining was quantified (**D**). Immunofluorescence analysis was performed to detect IL-6 in VAT sections from CD (**E**), HF + PA (**F**), and HF + LA-fed mice (**G**). The intensity of IL-6 staining was quantified (**H**). Values are expressed as mean ± SEM of 4–6 samples in each group. * *p* < 0.05 versus CD. MCP-1, monocyte chemoattractant protein-1; IL-6, interleukin-6; CD, chow diet; HF + PA, high fat + palmitic acid; HF + LA, high fat + lauric acid.

**Figure 3:**
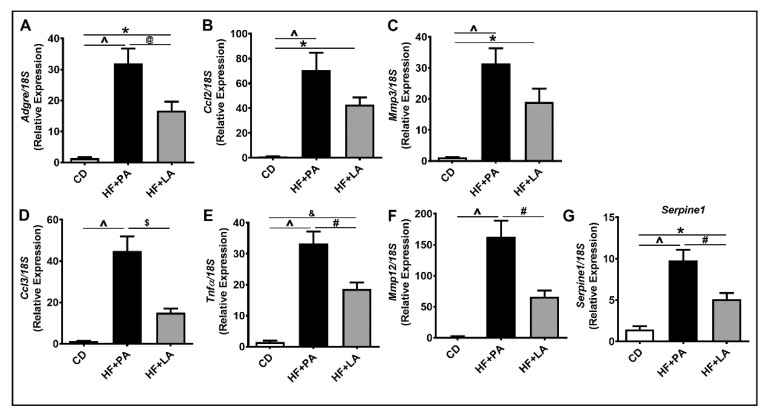
Effect of HF diet supplemented with PA or LA on the mRNA levels of inflammatory markers in VAT. Quantitative real-time PCR analysis was performed in the visceral adipose tissue (VAT) for the mRNA levels of *Adgre*, a macrophage marker (**A**) and other inflammatory genes (**B**–**G**). Values are expressed as mean ± SEM of 6–8 samples in each group. * *p* < 0.05, ^&^ *p* < 0.01, and ^ *p* < 0.001 versus CD; ^@^ *p* < 0.05, ^#^ *p* < 0.01, and ^$^ *p* <0.001 versus HF + PA. *Adgre1 (Emr-1)*, EGF-like module containing, mucin-like, hormone receptor like 1; *Ccl2,* C-C chemokine ligand 2; *Mmp3,* Matrix metallopeptidase 3; *Ccl3*, C-C chemokine ligand 3; *Tnf*α, Tumor necrosis factor, alpha; *Mmp12,* Matrix metallopeptidase 12; *Serpine1,* Serpin peptidase inhibitor, clade E; CD, chow diet; HF + PA, high fat + palmitic acid; HF + LA, high fat + lauric acid.

We next analyzed markers of inflammation in the VAT. IHC analysis of VAT sections for MCP-1, a marker of inflammation, showed a significant increase in MCP-1 staining only in HF + PA-fed mice but not in HF + LA-fed mice (Figure 2A–D). We next detected IL6 levels in AT by immunofluorescence. Similar to MCP-1, IL6 level was significantly higher only in HF + PA-fed mice but not in HF + LA-fed mice compared to CD controls (Figure 2E–H). Further analysis of markers of VAT inflammation by real-time PCR revealed that the mRNA level of *Adgre* (encoding F4/80), a marker of macrophages, was lower in HF + LA-fed mice compared to HF + PA-fed mice (Figure 3A). The mRNA level of C-C motif chemokine ligand 2 (*Ccl2*, encoding MCP-1) and Matrix metalloproteinase 3 (*Mmp3)* were significantly increased only in HF + PA-fed mice but not in HF + LA-fed mice compared to CD controls (*p* < 0.001) (Figure 3B,C). Further, the mRNA levels of C-C motif chemokine ligand 3 (*Ccl3*, encoding MIP1α), Tumor necrosis factor α (*Tnf*α), Matrix metalloproteinase 12 (*Mmp12)*, and Serpin Family E Member 1 (*Serpine 1,* encoding PAI-1) showed a profound increase in HF + PA-fed mice compared to CD controls. Interestingly, the expression of these genes was significantly lower in HF + LA-fed mice compared to HF + PA-fed mice (Figure 3D,G). Taken together, these data suggest that supplementation of HF diet with LA results in less AT inflammation compared to PA supplementation in mice.

### 3.3. Both PA and LA Supplementation Induced IR, But LA Was Less Potent Than PA in Causing IR Compared to CD Controls

**Figure 4:**
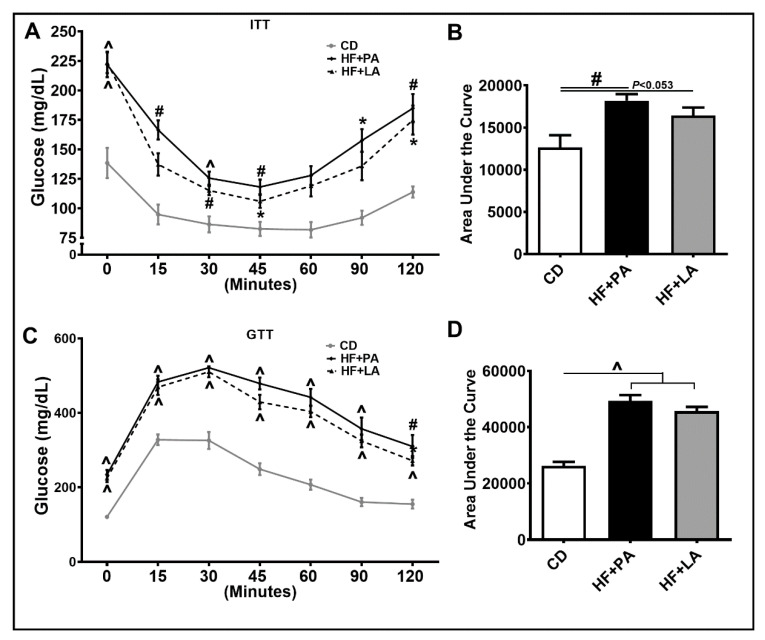
Effect of HF diet supplemented with PA or LA on systemic insulin resistance (IR). Mice were intraperitoneally injected with insulin for insulin tolerance test (ITT, **A**) and glucose for glucose tolerance test (GTT) (**C**) and blood glucose was recorded at indicated time points (**A**,**C**). Area under the curve glucose values from ITT (**B**) and GTT (**D**) were determined. Values are expressed as mean ± SEM of 6–8 samples in each group. * *p* < 0.05, ^#^ *p* < 0.01, and ^ *p* < 0.001 versus CD. CD, chow diet; HF + PA, high fat + palmitic acid; HF + LA, high fat + lauric acid.

AT inflammation is tightly linked to systemic IR [5]. We wanted to check whether LA and PA exert differential effects on IR as well. As shown in Figure 4A, ITT revealed that an HF + PA diet led to a pronounced increase in IR. However, the degree of IR was less in HF + LA-fed mice. The area under the curve (AUC) measurement showed a significant increase in HF + PA-fed mice (*p* <0.01) compared to CD controls. Although the HF + LA-fed mice did not show a significant increase in IR compared to CD controls, these mice showed a strong trend towards an increase in IR (*p* <0.053) (Figure 4B). Furthermore, GTT on these mice showed impaired glucose tolerance in both HF + PA and HF + LA-fed mice compared to CD controls (Figure 4C,D).

**Figure 5:**
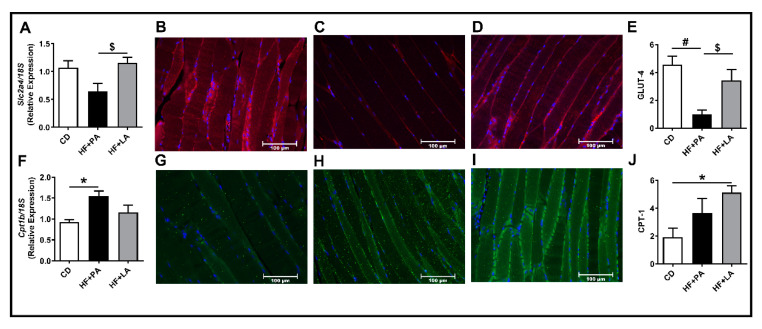
Effect of HF diet supplemented with PA or LA on markers of glucose and fatty acid transport in skeletal muscle. Quantitative real-time PCR analysis was performed in the skeletal muscle for the mRNA levels of genes involved in glucose and fatty acid transport/oxidation (**A**,**F**). Immunofluorescence analysis was carried out to determine the protein levels of GLUT-4 (**B**–**E**) and CPT-1 (**G**–**J**). Representative images at 20× magnification are shown. The intensity of staining was quantified and values are expressed as mean ± SEM of 6–8 samples in each group. * *p* < 0.05 and ^#^ *p* < 0.01 versus CD; ^$^ *p* < 0.05 versus HF + PA. Slc2a4 (GLUT-4), Solute carrier family 2 (facilitated glucose transporter), member 4; Cpt1b, Carnitine palmitoyltransferase 1b; CD, chow diet; HF + PA, high fat + palmitic acid; HF + LA, high fat + lauric acid.

Because systemic IR was increased to a lesser extent in HF + LA-fed mice compared to HF + PA-fed mice, we next wanted to see whether PA and LA exert differential effects on substrate metabolism in skeletal muscle. Therefore, we next analyzed markers of glucose and fatty acid transport and metabolism. Real-time PCR analysis revealed that the mRNA level of Solute Carrier Family 2 Member 4 (*Slc2a4*, encoding Glucose transporter 4 or GLUT4) was significantly higher in HF + LA-fed mice compared to HF + PA-fed mice (Figure 5A). Further immunofluorescence analysis of skeletal muscle showed that GLUT-4 protein was significantly lower in HF + PA-fed mice whereas the GLUT-4 level was higher in HF + LA-fed mice compared to HF + PA-fed mice (Figure 5B–E). The mRNA level of Carnitine palmitoyl transferase 1b (*Cpt1b*), a marker of skeletal muscle mitochondrial fatty acid transport and oxidation, was significantly higher in HF + PA-fed mice but not in HF + LA-fed mice compared to CD controls (Figure 5F). However, CPT-1 protein level was significantly increased only in HF + LA-fed mice but not in the HF + PA-fed group (Figure 5G–J). Taken together, these data suggest that a reduction in AT inflammation and improvements in skeletal muscle glucose and fatty acid oxidation may play a role in partly mediating the effects of LA in lowering IR to some extent.

### 3.4. LA Supplementation Caused Less Liver Injury Compared to PA

**Figure 6:**
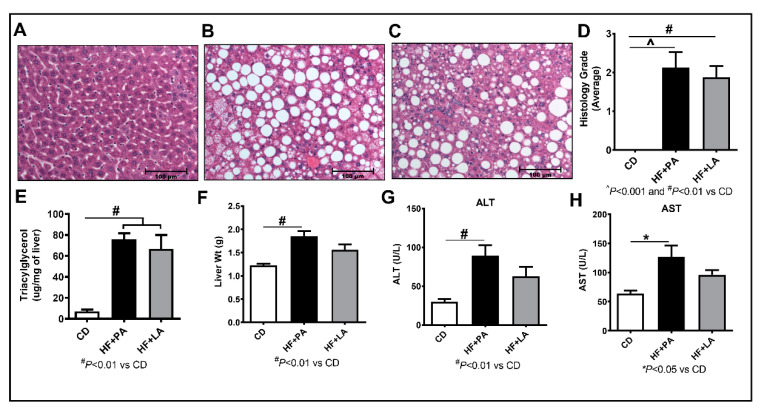
Effect of HF diet supplemented with PA or LA on markers of non-alcoholic fatty liver disease (NAFLD). Liver sections (4 µm) from CD (**A**), HF + PA (**B**), and HF + LA-fed mice (**C**) were stained with hematoxylin and eosin. Pictures were taken at 20X magnification using a Nikon Eclipse 80i microscope. Histological grade for the degree of steatosis is shown (**D**). Quantification of liver triacylglycerol by gas chromatography (**E**). Liver weight measured at sacrifice is provided (**F**). Plasma levels of ALT and AST, markers of liver injury are shown (**G**,**H**). Values are expressed as mean ± SEM of 6–8 samples in each group. * *p* < 0.05, ^#^ *p* < 0.01, and ^ *p* < 0.001 versus CD. ALT, alanine aminotransferase; AST, aspartate aminotransferase; CD, chow diet; HF + PA, high fat + palmitic acid; HF + LA, high fat + lauric acid.

A HF diet feeding leads to the development of NAFLD. To determine whether LA and PA lead to differential effects on NAFLD, we first performed H&E staining of liver sections, which showed a significant increase in steatosis in HF + PA (*p* < 0.001) and HF + LA (*p* < 0.01)-fed mice compared to CD controls (Figure 6A–D). The extent of steatosis in HF + LA-fed mice was slightly less compared to HF + PA-fed mice. While all the HF + PA and HF + LA-fed mice showed some level of steatosis, only three mice from each group showed inflammatory nodules. Moreover, only one mouse in each of these groups showed a ballooning phenotype and none of the mice showed fibrosis. Taken together, histological markers of liver injury did not differ much between HF + PA and HF + LA-fed mice. Further, we measured liver triacylglycerol by gas chromatography and noted that hepatic triacylglycerol levels were significantly higher in both HF + PA- and HF + LA-fed mice (*p* < 0.01) compared to CD controls. However, the triglyceride level showed a small reduction in HF + LA-fed mice compared to HF + PA-fed group. We noted a 12% reduction in liver triacylglycerol level in HF + LA-fed mice compared to HF + PA-fed mice (Figure 6E). Moreover, the total liver weight was significantly higher only in HF + PA-fed mice (*p* < 0.01) but not in HF + LA-fed mice compared to CD controls (Figure 6F). Analysis of systemic markers of liver injury revealed a significant increase in plasma levels of ALT (*p* < 0.01) and AST (*p* < 0.05) only in HF + PA-fed mice but not in HF + LA-fed mice (Figure 6G,H). These data suggest that the MCSFAs, in particular, LA, attenuates markers of liver injury in mice at least to some extent.

### 3.5. A High Fat Diet Supplemented with LA or PA Exerted Similar Effects on Fatty Acid Profile in Liver Triglycerides

To determine whether supplementation with PA or LA leads to the incorporation of respective fatty acids in the liver, we analyzed the fatty acid profile in liver triglycerides. As shown in Table 2, the levels of all the major fatty acids are increased significantly in both HF + PA and HF + LA-fed mice. Of note, while PA was increased significantly in both HF + PA and HF + LA-fed mice compared to CD controls, it was increased to a greater extent in HF + PA-fed mice (*p* < 0.001) than in HF + LA-fed mice (*p* < 0.01). On the other hand, LA was undetectable in liver samples even in HF + LA-fed mice, likely due to rapid modification of this fatty acid in the liver.

### 3.6. Hepatic Oxidative Stress Was Not Altered by a HF Diet Supplemented with LA or PA

**Figure 7:**
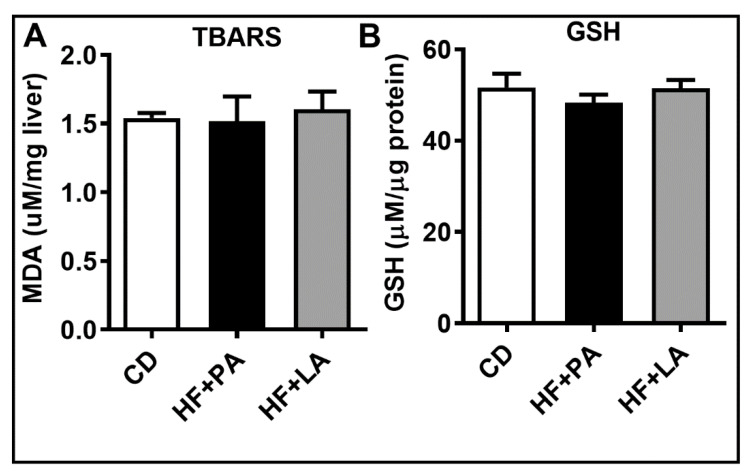
Effect of HF diet supplemented with PA or LA on markers of hepatic oxidative stress. The degree of lipid peroxidation was assessed through measurement of TBARS (**A**). Hepatic levels of total GSH are shown (**B**). Values are expressed as mean ± SEM of 6–8 samples in each group. MDA, malondialdehyde; TBARS, thiobarbituric acid reactive substances; GSH, glutathione; CD, chow diet; HF + PA, high fat + palmitic acid; HF + LA, high fat + lauric acid.

To determine whether PA and LA exert differential effects on hepatic oxidative stress, we next measured TBARS levels and GSH content in the liver. We noted that these markers were not altered significantly among different groups (Figure 7).

### 3.7. A High Fat Diet Supplemented with LA or PA Exerted Similar Effects on Markers of Hepatic Inflammation

**Figure 8:**
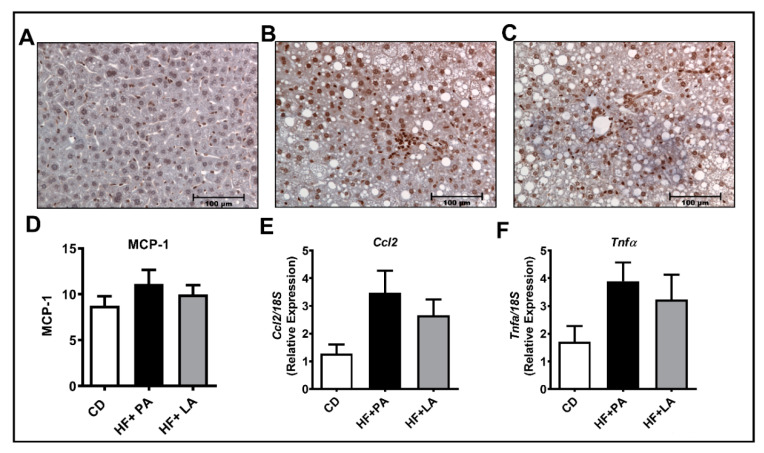
Effect of HF diet supplemented with PA or LA on markers of hepatic inflammation. Immunohistochemical analysis was performed to detect MCP-1 in liver sections from CD (**A**), HF + PA (**B**), and HF + LA-fed mice (**C**). Representative images at 20× magnification are shown. The intensity of MCP-1 staining was quantified (**D**). Quantitative real-time PCR was performed for the mRNA levels of inflammatory genes such as *Ccl2* and *Tnf*α (**E**,**F**). Values are expressed as mean ± SEM of 6–8 samples in each group. MCP-1, monocyte chemoattractant protein-1; *Ccl2*, C-C chemokine ligand 2; *Tnf*α, Tumor necrosis factor α; CD, chow diet; HF + PA, high fat + palmitic acid; HF + LA, high fat + lauric acid.

We next wanted to check whether the markers of hepatic inflammation were altered among groups. Of note, the IHC analysis for MCP-1 protein did not show a significant difference among different groups of mice (Figure 8A–D). Moreover, the real-time PCR analysis showed that the mRNA levels of inflammatory genes such as *Ccl2* and *Tnf*α did not change significantly among different groups (Figure 8E,F). The mRNA levels of other inflammatory genes including *Il6*, *Ccl3*, and *Mmp3* were not altered among different groups (not shown). Western blot analysis further showed no change in MCP-1, MMP7, and MMP12 protein levels (not shown) and TNFα and IL6 were undetectable. Overall, we did not notice a considerable increase in markers of hepatic inflammation in HF + LA and HF + PA-fed mice compared to CD controls.

## 4. Discussion

LA is the most abundant SFA in coconut oil, which is known to exert beneficial metabolic effects against hepatic steatosis, IR, and cardiovascular disease [11,12,15,16,17,18]. However, concerns exist regarding the benefit of using coconut oil against cardiovascular disease [19,20]. Of note, the effect of purified LA in altering AT inflammation and other metabolic disorders in obesity remains unknown. Such studies are important to better assess the biological effects of individual SFAs. Using an HF diet supplemented with purified PA or LA, we have demonstrated that PA and LA exert differential effects in AT, muscle, and liver. LA increased VAT mass to a greater extent than PA. However, markers of AT inflammation were lower in mice fed an LA supplemented HF diet compared to those receiving PA in the diet. Consistent with its effects in suppressing AT inflammation, LA also exerted less systemic IR. Interestingly, markers of muscle glucose and fatty acid oxidation are higher in the skeletal muscle of HF + LA-fed mice. Plasma markers of liver injury, in particular, ALT and AST, were increased to a lesser extent in mice fed an LA-supplemented diet compared to PA. Taken together, our data suggest that LA exerts less deleterious metabolic effects in AT, muscle, and liver compared to PA and that LA may mediate some of the metabolic benefits of coconut oil.

Most of the studies evaluating the effects of MCSFAs and LCSFAs used a diet enriched in coconut oil or palm oil, respectively [11,22,23,24,25]. Very little is known regarding the specific effect of LA in altering metabolic processes in vivo. For example, a 1% LA supplemented diet promoted glycolytic muscle formation in mice [26]. In another study, LA supplementation of control diet at 1% promoted the lactation function of mice [27]. It should be noted that these studies used a normal diet enriched with LA. On the other hand, a study in rats fed 20% of individual SFAs including LA and PA, body weight gain and adiposity was lower in LA-supplemented diet compared to mice fed a diet containing PA [28]. However, the specific effect of purified LA supplementation in a HF diet in altering obesity-related AT inflammation and NAFLD remains unclear.

One of the findings of this study is that LA increased VAT mass but exerted less AT inflammation compared to PA. The primary function of AT is to store excess fat in fed conditions and to release FFA and glycerol in the fasted conditions. To check whether increased VAT mass in HF + LA-fed mice could be due to a reduction in AT lipolysis, we checked basal and stimulated lipolysis in HF + PA- and HF + LA-fed groups. We did not see a significant difference in basal or stimulated lipolysis between HF + PA- and HF + LA-fed mice. Instead, we noted an increase in plasma glycerol levels, a marker of AT lipolysis, in HF + LA-fed mice compared to HF + PA-fed mice. These data indicate that LA does not impair the release of FFAs in the fasted condition and that the increase in VAT mass could be due to efficient storage of excess FFAs in the fed condition. Although an increased VAT mass is often associated with an increase in AT inflammation, we noted that markers of AT inflammation are significantly lower in LA-fed mice compared to PA-fed mice. Thus, it appears reasonable to speculate that different fatty acids have differential effects on VAT lipid storage function and that an increase in VAT mass, if not associated with VAT inflammation, may actually improve systemic metabolic homeostasis.

Obesity is often associated with macrophage infiltration and inflammation in AT [29]. LCSFAs, in particular, PA, is known to promote an inflammatory response in both macrophages as well as adipocytes [30,31,32,33]. Accordingly, we noted that markers of inflammation are profoundly increased in the AT of HF + PA-fed mice. On the other hand, markers of AT inflammation are much lower despite increased VAT mass in mice fed an HF + LA diet. We previously showed that fish oil, which contains ω-3 polyunsaturated fatty acids, exerted similar effects in VAT [34]. In this study, feeding mice a fish oil diet increased VAT mass but reduced VAT inflammation. Coconut oil has been shown to differentially alter adiposity and AT inflammation. For example, coconut oil increased adiposity and AT inflammation in C57BL/6J mice [35]. In addition, virgin coconut oil supplementation increased adipocyte hypertrophy and promoted AT inflammation in rats [36]. On the other hand, in another study, virgin coconut oil has been shown to reduce adiposity and AT inflammation in BALB/c mice fed a high-refined carbohydrate-containing diet [12]. The varied effects of coconut oil in different studies could be due to the experimental conditions. For example, coconut oil is effective in reducing adiposity and AT inflammation in high carbohydrate diet-fed mice [12]. However, when coconut oil was supplemented in a HF diet, it promoted adiposity and AT inflammation [35]. As mentioned, coconut oil not only contains LA but also certain LCSFAs such as myristic acid and PA, which promote inflammation. Our study provides clear evidence that an HF diet supplemented with LA alone, the predominant SFA found in coconut oil, increases VAT mass without altering total adiposity and exerts less AT inflammation compared to a PA-enriched diet.

Another interesting observation is that IR was increased to a lesser extent in HF + LA-fed mice compared to HF + PA-fed mice. LCSFAs, in particular, PA, are known to promote IR (reviewed in [37]). However, a diet enriched in coconut oil rich in MCSFAs, led to a decrease or no change in IR. For example, Zacek et al. have shown that an HF diet supplemented with coconut oil reduced IR in mice [11]. In other studies, coconut oil as a fat source in diet did not promote IR in rodents and humans [38,39,40]. Our data show that specific LA supplementation caused less IR compared to PA supplementation in diet, further suggesting that MCSFAs have a favorable effect in controlling metabolic disorders compared to LCSFAs. As for potential mechanisms, AT inflammation, which promotes systemic IR is attenuated by the LA diet. In addition, the protein level of GLUT-4, a marker of glucose transport, was significantly lower in HF + PA-fed mice and not altered in HF + LA-fed mice compared to CD controls. Moreover, CPT-1, a marker of fatty acid transport, was higher in HF + LA-fed mice, indicating that skeletal muscle substrate metabolism is higher in HF + LA-fed mice compared to HF + PA-fed mice. These effects are consistent with a previous report showing that the expression of both GLUT-4 and CPT-1 are increased in myotubes upon LA treatment [41]. Further, an LA supplemented diet promoted glycolytic muscle formation in mice [26].

Regarding the effect of LA versus PA on liver functions, we noted that the liver weight is significantly higher only in HF + PA-fed mice but not in HF + LA-fed group compared to CD controls. Moreover, plasma levels of ALT and AST, markers of liver injury, are significantly higher in mice receiving the PA-containing diet but not in those fed the LA-enriched diet, suggesting that LA was effective in blunting HF diet-induced impairments in liver function. Steatosis was increased in both HF + PA and HF + LA-fed mice and not much difference was noted between the two groups.

It is interesting to note that AT inflammation is increased by HF + PA-fed mice whereas hepatic inflammation was not significantly altered in our study. It should be pointed out that AT inflammation is preceded by hepatic inflammation upon an HF diet feeding. For example, a study by Stanton et al. showed that mice when fed an HF diet showed an increase in AT inflammation but not hepatic inflammation after 6 wk of HF feeding. However, when the feeding period was increased to 26 wk, mice exhibited a profound increase in liver inflammation [42], indicating that a lack of a considerable increase in hepatic inflammatory markers in our study could be due to our experimental condition. First, we fed mice the experimental diet for only 12 wk at which time point, we noted a significant increase in AT inflammation but not a distinct increase in hepatic inflammation. Next, we used a modified HF diet where a portion of lard is replaced by PA or LA. Nevertheless, there is a trend towards an increase in mRNA and protein markers of inflammation in HF + PA-fed mice compared to CD controls. Studies in mice fed HF + PA and HF + LA diets for a longer time may provide a better idea on the effect of HF + LA in altering hepatic inflammation.

Taken together, our findings emphasize the beneficial metabolic effects of coconut oil, which contains ~55% LA, against obesity-related pathologies. In addition to improving metabolism, LA has potent anti-microbial [43,44] and anti-cancer [45,46] properties. Although coconut oil is known to have cardio-protective effects, concerns exist regarding the benefit of this oil in protecting against heart diseases [19,20]. The adverse effects of coconut oil reported in some studies could be due to the presence of other LCSFAs. It should be pointed out that the metabolic benefits of coconut oil may also be due, in part, to the other MCFAs such as caprylic acid and capric acid. Therefore, future studies are warranted to determine the role of these fatty acids on obesity-associated metabolic disorders.

## 5. Conclusions

Taken together, our findings suggest that LA is less deleterious than PA with regard to obesity-related metabolic disorders. Our findings highlight the importance of supplementing purified fatty acids as opposed to dietary oils to determine the role of specific SFAs in altering obesity-linked metabolic disorders in vivo.

## Figures and Tables

**Table 1:** Primers used in the study.

Gene (Abbr)	Description	Catalog Number
*18s*	18S ribosomal RNA	4352930E
*Adgre1 (EMR-1; F4/80)*	EGF-like module containing, mucin-like, hormone receptor-like 1	Mm00802529_m1
*Ccl2 (MCP-1)*	Chemokine ligand 2/monocyte chemotactic protein 1	Mm00441242_m1
*Ccl3 (MIP-1a)*	Chemokine ligand 3/macrophage inflammatory protein 1alpha	Mm00441258_m1
*Cpt1b*	Carnitine palmitoyl transferase 1 (A = Liver; B = Muscle)	Mm00487191_g1
*Cpt2*	Carnitine palmitoyl transferase 2	Mm00487205_m1
*Mmp12*	Matrix metallopeptidase 12 (macrophage elastase)	Mm00500554_m1
*Mmp3*	Matrix metallopeptidase 3	Mm00440295_m1
*Serpine/PAI-1*	Serpin peptidase inhibitor, clade E (nexin, plasminogen activator inhibitor type 1)	Mm00435860_m1
*Slc2a4 (Glut-4)*	Solute carrier family 2 (facilitated glucose transporter), member 4	Mm01245502_m1
*Tnfa*	Tumor necrosis factor alpha	Mm00443258_m1

**Table 2:** Fatty acid profile in liver triglycerides.

Fatty Acid	CD	HF + PA	HF + LA
Myristic acid (14:0)	0.02 ± 0.02	0.59 ± 0.04 ^	0.61 ± 0.11 ^
Palmitic acid (16:0)	2.66 ± 0.50	24.7 ± 1.9 ^	21.0 ± 4.3 ^#^
Palmitoleic acid (16:1)	0.26 ± 0.06	3.28 ± 0.24 ^	3.05 ± 0.64 ^
Stearic acid (18:0)	0.28 ± 0.05	1.38 ± 0.11 ^	1.18 ± 0.18 ^
Oleic acid (18:1)	2.86 ± 0.56	29.2 ± 2.6 ^	26.7 ± 5.5 ^#^
Linoleic acid (18:2)	2.10 ± 0.56	10.7 ± 0.7 ^	9.54 ± 1.50 ^

Fatty acid levels are expressed as µg/mg tissue. Values are mean ± SEM of 6–8 samples per group. ^#^ *p* < 0.01 and ^ *p* < 0.001 versus CD. CD, chow diet; HF, high fat; PA, palmitic acid; LA, lauric acid.
